# Evidence of primary cilia in the developing rat heart

**DOI:** 10.1186/s13630-018-0058-z

**Published:** 2018-07-31

**Authors:** Sarbjot Kaur, Sue R. McGlashan, Marie-Louise Ward

**Affiliations:** 1 Department of Physiology, School of Medical Sciences, Faculty of Medical and Health Sciences, The University of Auckland, Private Bag 92019, Auckland, 1023 New Zealand; 2 Department of Anatomy and Medical Imaging, School of Medical Sciences, Faculty of Medical and Health Sciences, The University of Auckland, Auckland, New Zealand

**Keywords:** Primary cilia, ARL13B, Rat cardiac tissue, Cardiac trabeculae, Isolated cardiomyocytes

## Abstract

**Background:**

A transient increase in cytosolic Ca^2+^ (the “Ca^2+^ transient”) determines the degree and duration of myocyte force development in the heart. However, we have previously observed that, under the same experimental conditions, the Ca^2+^ transients from isolated cardiac myocytes are reduced in amplitude in comparison to those from multicellular cardiac preparations. We therefore questioned whether the enzymatic cell isolation procedure might remove structures that modulate intracellular Ca^2+^ in some way. Primary cilia are found in a diverse range of cell types, and have an abundance of Ca^2+^-permeable membrane channels that result in Ca^2+^ influx when activated. Although primary cilia are reportedly ubiquitous, their presence and function in the heart remain controversial. If present, we hypothesized they might provide an additional Ca^2+^ entry pathway in multicellular cardiac tissue that was lost during cell isolation. The aim of our study was to look for evidence of primary cilia in isolated myocytes and ventricular tissue from rat hearts.

**Methods:**

Immunohistochemical techniques were used to identify primary cilia-specific proteins in isolated myocytes from adult rat hearts, and in tissue sections from embryonic, neonatal, young, and adult rat hearts. Either mouse anti-acetylated α-tubulin or rabbit polyclonal ARL13B antibodies were used, counterstained with Hoechst dye. Selected sections were also labelled with markers for other cell types found in the heart and for myocyte F-actin.

**Results:**

No evidence of primary cilia was found in either tissue sections or isolated myocytes from adult rat ventricles. However, primary cilia were present in tissue sections from embryonic, neonatal (P2) and young (P21 and P28) rat hearts.

**Conclusion:**

The lack of primary cilia in adult rat hearts rules out their contribution to myocyte Ca^2+^ homoeostasis by providing a Ca^2+^ entry pathway. However, evidence of primary cilia in tissue from embryonic and very young rat hearts suggests they have a role during development.

**Electronic supplementary material:**

The online version of this article (10.1186/s13630-018-0058-z) contains supplementary material, which is available to authorized users.

## Background

Intracellular Ca^2+^ has a crucial role in the heart’s contraction, directly controlling the force developed by the myocytes with every heart “beat”. Cyclical changes in intracellular Ca^2+^ are initiated by the cardiac action potential, known as the “Ca^2+^ transient”, and form the basis of excitation–contraction coupling in the heart (for review see [1]). Ca^2+^ homoeostasis within myocytes is therefore of major importance to the function of the heart, since all myocytes contribute to every heartbeat. Small alterations in the amplitude and/or the time course of the Ca^2+^ transient are immediately reflected in the force developed during subsequent contraction. Previously, we have observed that isolated myocytes were depotentiated, with low-amplitude Ca^2+^ transients in comparison to those from multicellular ventricular trabeculae under the same experimental conditions [2]. A major difference between isolated myocytes and intact cardiac preparations, such as trabeculae, is the loss of the extracellular matrix during the cell isolation process. We therefore questioned whether transmembrane spanning structures that modulate intracellular Ca^2+^ in other cell types, such as primary cilia [3], might also have a role in the heart. We hypothesized that the primary cilia may be lost during the enzymatic myocyte isolation process, leading to a net loss of intracellular [Ca^2+^] and reduced Ca^2+^ transients.

The primary cilium is a solitary, non-motile organelle that is an extension of the basal body and is ubiquitously expressed in mammalian cell types [4, 5]. As sensory organelles, they transduce external forces, perhaps via intracellular Ca^2+^ signals [6] (but see [7]), coordinating multiple signalling pathways [8–13]. Defects lead to several human diseases collectively known as ‘ciliopathies’ [14, 15, reviewed in 21, 22], with instances of impaired left ventricular function and diffuse interstitial fibrosis of the heart [16]. Primary cilia are reportedly important in the development of a number of organs [17, 18]. However, primary cilia in adult cardiac myocytes, and their possible contribution to intracellular [Ca^2+^]_i_ modulation, have not been fully investigated.

The aim of this study was therefore to look for the evidence of primary cilia in situ in young and adult ventricular tissue, and in isolated myocytes, from rat hearts through immunohistochemistry and confocal microscopy.

## Methods

Methods relating to the cardiac myocyte isolation procedure are provided in the Additional file 1, together with details of the solutions, reagents, and chemicals used.

## Results

### Immunolabelling in adult single cardiomyocytes and cardiac tissue

**Fig. 1:**
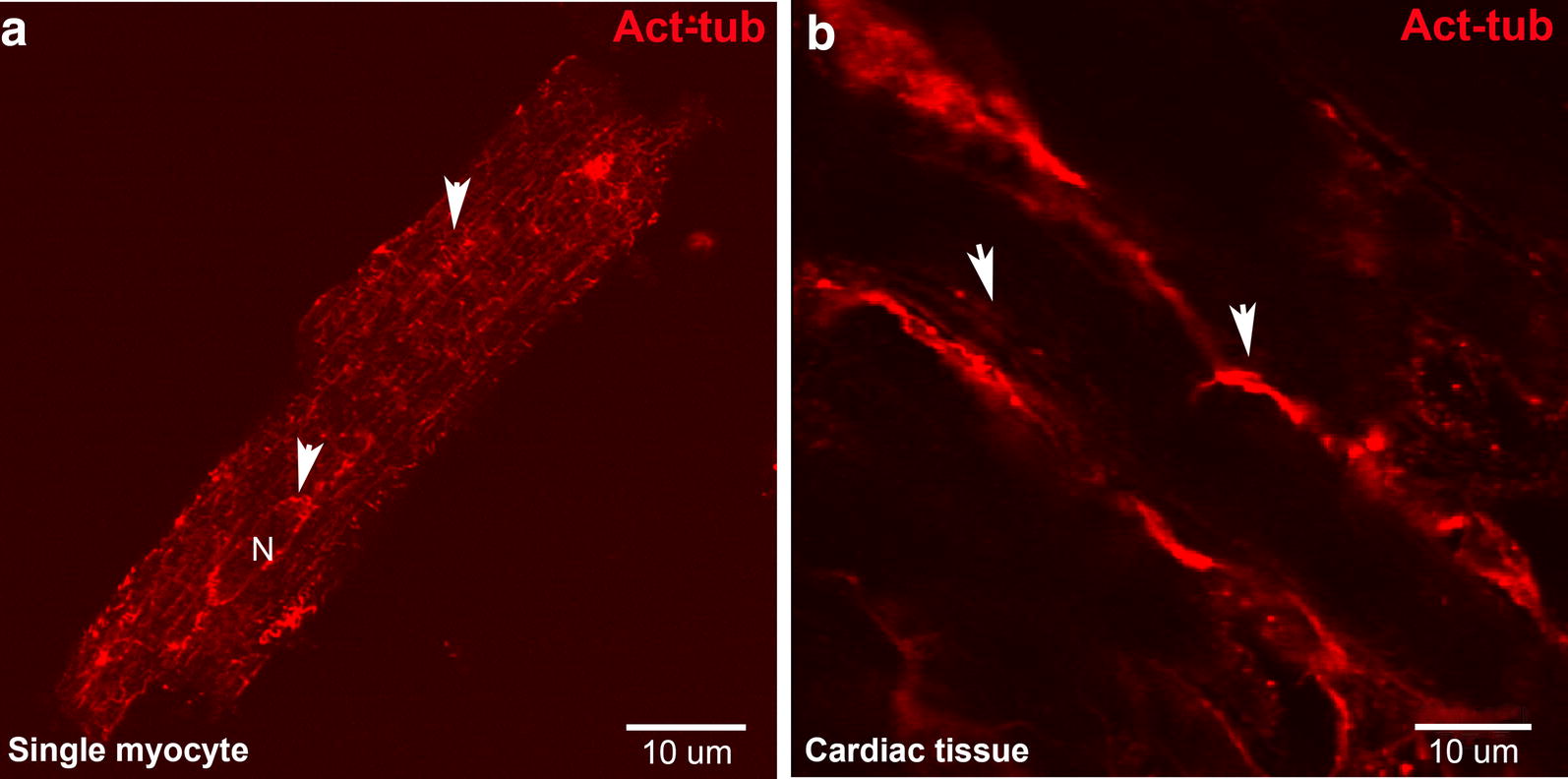
Acetylated-α-tubulin in single myocyte and cardiac tissue. Confocal images of **a** a representative adult rat cardiomyocyte and **b** adult rat ventricular tissue, immunolabelled for acetylated α-tubulin. ‘N’ shows the nucleus. A fine network of cytoplasmic tubulin labelling is present in **a**, with dense labelling close to the nuclear regions shown by arrows. Arrowheads indicate intense tubulin labelling in between myocytes in extracellular spaces in the representative cardiac tissue section shown in **b**

**Fig. 2:**
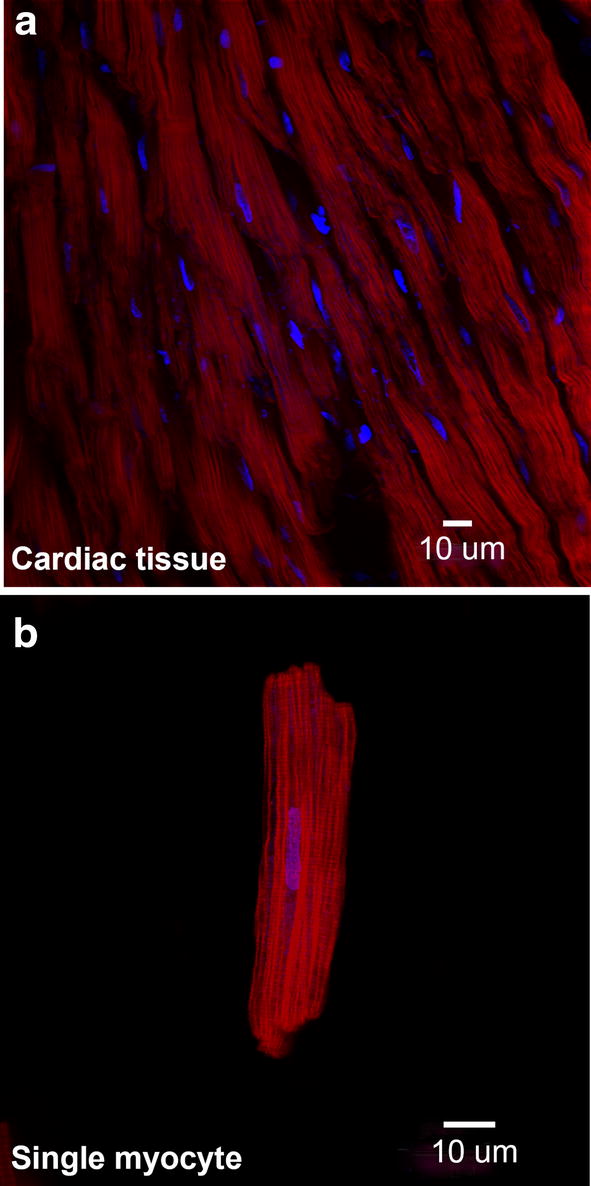
Absence of ARL13B labelling in adult tissue and single myocytes. Representative confocal images dual immunolabelled for ARL13B (red) and nuclei (blue). **a** Adult rat cardiac tissue and **b** adult rat cardiomyocyte. A scale bar is shown for each image. ARL13B labelling was completely absent from both adult rat cardiac tissue and cardiomyocytes. A high background autofluorescence at lower intensity shows tissue and myocyte structure, with no ARL13B antibody labelling

Figure 1a shows a representative confocal image taken at the surface of a mature isolated myocyte labelled for acetylated-α-tubulin. A very bright region of labelling near the nucleus was observed in all cells (20–50 cells, from *n* = 2 hearts). A fine network of positively stained microtubules was also seen within all of the myocytes, showing that the acetylated-α-tubulin antibody labels cytoplasmic tubulin, and not just primary cilia. In rat ventricular tissue sections, intense staining of the extracellular spaces between myocytes, along with some intracellular labelling, was also observed (Fig. 1b; see Additional file 2: Figure S2 for details). These results showed that acetylated α-tubulin was not a reliable primary antibody for identification of primary cilia in adult rat tissue. Therefore, ARL13B was used in further experiments [19–21]. Kidney sections from adult rats were also labelled with, and without, primary antibodies (anti-acetylated α-tubulin/anti-ARL13B) to provide positive and negative controls (Additional file 3: Figure S1). Confocal images showed no evidence of primary cilia in rat tissue sections (*n* = 4 hearts, with at least five sections from each heart), or in isolated myocytes (*n* = 4 hearts, 20–50 cells per heart) from adult (~ 2 months of age) rat hearts, with representative images shown in Fig. 2.

### Immunolabelling in embryonic, neonatal, and young rat cardiac tissue

**Fig. 3:**
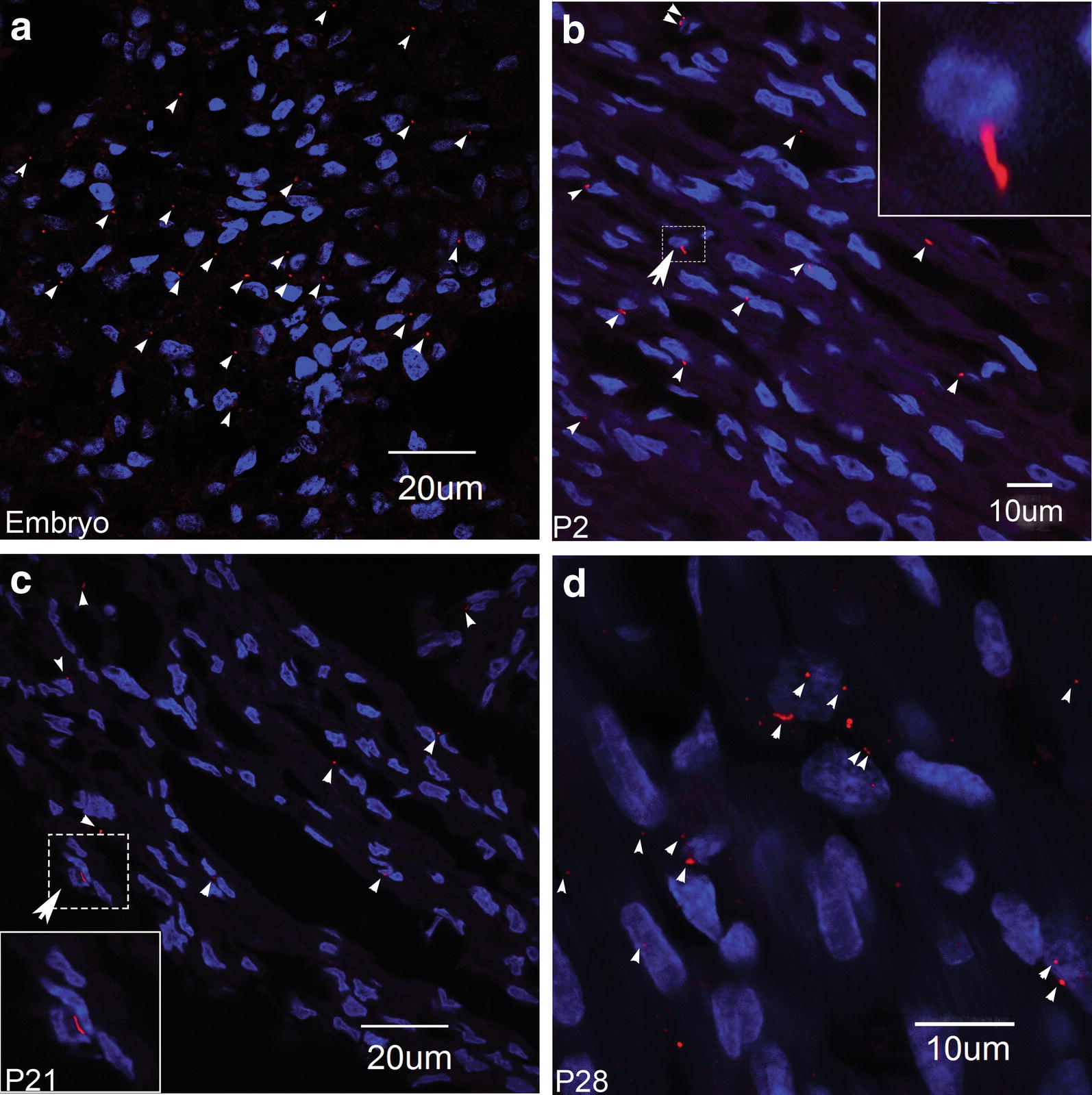
ARL13B labelling of cardiac tissue from embryonic, neonatal, and young rat hearts. Confocal images immunolabelled for ARL13B (red) and nuclei (blue) in **a** embryonic, **b** neonatal (P2), and **c**, **d** young (P21 and P28) rat cardiac tissues. The small arrowheads show the presence of primary cilia in these images. Insets: magnified views of the primary cilia and nucleus from P2 and P21 rat cardiac tissue sections from the regions indicated by the large arrowheads

Tissue sections from embryonic (*n* = 3), neonatal (P2, *n* = 2), and young (P21 and P28, *n* = 3 each) rat hearts were immunolabelled for primary cilia with anti-ARL13B antibody (Fig. 3a–d). Primary cilia were present in sections from embryonic, neonatal, and young cardiac tissue, which were located close to the nucleus of most cells. The distribution of primary cilia per nucleus appeared to be more abundant in embryonic sections in comparison to sections from neonatal and P2–P28 hearts.

### Primary cilia association to cell type in neonatal rat cardiac tissue

**Fig. 4:**
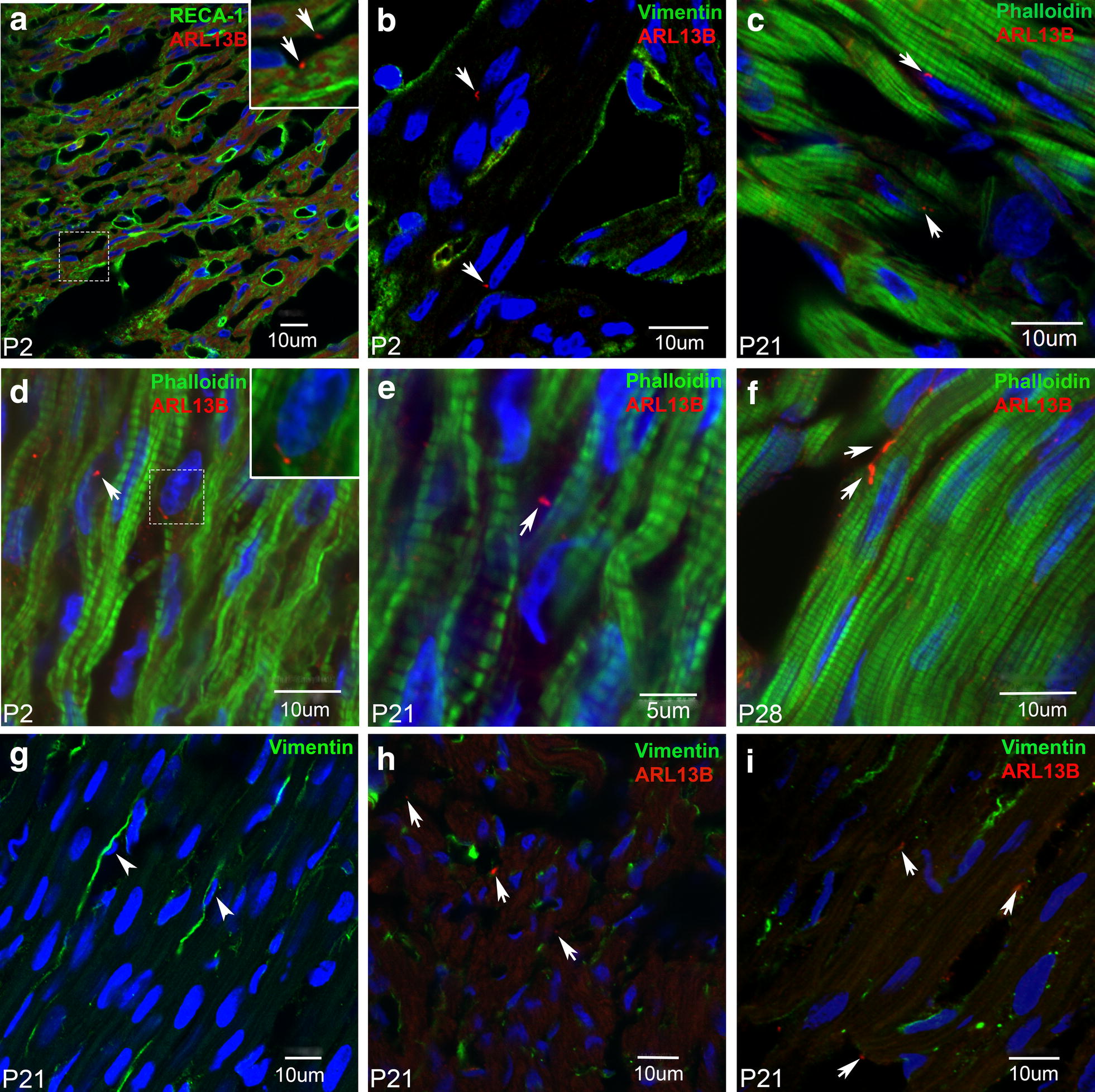
Association of ARL13B with cell type in neonatal cardiac tissue. Representative confocal images of neonatal rat cardiac tissue sections triple immunolabelled with Hoechst (blue), ARL13B in red; with RECA-1 (**a**), vimentin (**b**), or phalloidin (**c**–**f**) labels in green. Cilia, identified by white arrows in each image, were not associated with either endothelial cells (**a**) or with interstitial fibroblasts (**b**, **h**, **i**, vimentin). ARL13B-positive structures always appeared to be associated with myocyte f-actin (**c**–**f**, phalloidin). White arrowheads (**g**) show fibroblasts with nuclei which are much smaller in size than the more abundant myocyte nuclei. Insets in **a** and **d** show magnified views of the dotted boxes

Results showed primary cilia labelling was not associated with either endothelial cells (RECA-1 labelling, Fig. 4a) or fibroblasts (vimentin labelling, Fig. 4b, h and i) as shown in representative confocal images. Investigation of 3 × 10 µm tissue sections from P2, P21, and P28 rat hearts showed that primary cilia were not associated with fibroblasts (identified by vimentin labelling, and their smaller nuclei, Fig. 4g). Instead, the primary cilia were closely associated with myocyte contractile protein f-actin labelling in tissue from all three postnatal stages, as shown in Fig. 4c–f.

## Discussion

Previously, acetylated α-tubulin-based approaches have been used as evidence of primary cilia from immunofluorescence. However, this antibody labels cytoplasmic microtubules, as well as defined microtubule structures, such as primary cilia [19, 22–24]. Acetylated α-tubulin antibody also labels the tubulin associated with neurons, which are abundant in the heart [25]. We therefore utilized a more specific antibody for primary cilia which is a marker of the ARL13B protein, a small ciliary GTPase protein localized in the axonemal portion of the primary cilium and required for cilium biogenesis and maintenance [26, 27], and confirmed the presence of primary cilia in tissue from embryonic and developing rat hearts. Primary cilia are structures only 200–300 nm in diameter, and a few microns in length, making it technically difficult to capture them in a single plane in our images [28]. Hence, we include a video of Z-stacks from a positive control with primary cilia shown in different planes (Additional file 3: Figure S1(ii)). The specificity of anti-ARL13B antibody for primary cilia in the cardiovascular system has recently been reported in developing cardiomyocytes in culture [19], and in cultured endothelial cells [29].

We found no evidence of ARL13B labelling in either cardiac tissue sections, or isolated myocytes, from adult rat hearts (Fig. 2). This was surprising, since primary cilia have previously been reported in adult human hearts, identified by electron microscopy [30]. In contrast, we observed ARL13B labelling of primary cilia in tissue sections from embryonic, neonatal (P2) and young (P21 and P28) rat hearts (Fig. 3a–d). The ARL13B labelling was associated with the larger myocyte nuclei and f-actin myofilament protein (Fig. 4c–f).

## Conclusion

We showed primary cilia are present in the early stages of rat heart development, but are missing from the myocardium of mature rat hearts. Primary cilia cannot therefore explain the observed differences in Ca^2+^ homoeostasis between isolated myocytes and multicellular cardiac preparations from rat hearts.

## Additional files

**Additional file 1.** Methods relating to the cardiac myocyte isolation procedure together with details of the solutions, reagents, and chemicals used.

**Additional file 2.** **Acetylated-α-tubulin labelling in adult cardiac tissue.** A series of Z-stacks showing intracellular and extracellular tubulin labelling by acetylated α-tubulin (red) in adult rat cardiac tissue. Regions of intense staining are shown in the extracellular space, along with some less prominent intracellular staining of microtubules, illustrating the inappropriateness of acetylated-α-tubulin for identification of primary cilia in the heart. The scale bar is 10 µm.

**Additional file 3.** **Controls for acetylated α-tubulin and ARL13B antibodies.** (i) Confocal images of rat kidney sections taken from glomerulus and tubule regions. **A** Negative, and **B** positive controls for acetylated-α-tubulin. **C** Negative, and **D** positive controls for ARL13B. The images show the presence of primary cilia in the glomerulus region of the kidney. Scale bars are shown for each image. (ii) A video of Z-stacks from the rat kidney glomerulus region stained with acetylated-α-tubulin as a positive control show primary cilia in different planes.

## Abbreviations

[Ca^2+^]_o_: extracellular calcium
[Ca^2+^]_i_: intracellular calcium
ARL13B: ADP ribosylation factor-like 13B
RECA-1: rat endothelial cell antigen-1
P2: postnatal day 2
P21: postnatal day 21
P28: postnatal day 28
