# Supplementary material for: Evidence of primary cilia in the developing rat heart
Source: Cilia. 2018 Jul 31;7:4. doi: 10.1186/s13630-018-0058-z (PMC6069708; doi:10.1186/s13630-018-0058-z)
Supplement: Supplementary file 1 — Additional file 1. Methods relating to the cardiac myocyte isolation procedure together with details of the solutions, reagents, and chemicals used. [file 13630_2018_58_MOESM1_ESM.docx]

**Additional Methods**

**2. Methods**

*2.1 Myocyte isolation*

Ventricular myocytes were enzymatically-isolated from Wistar rat (200–300 g) hearts [1], using protocols approved by the University of Auckland Animal Ethical Committee. The rats were anaesthetized with isoflurane using 100% O_2_ as a carrier gas prior to decapitation. The heart was quickly dissected out and mounted by the aorta onto a Langendorff perfusion system. Initially, hearts were perfused with oxygenated Ca^2+^ -free Tyrode’s buffer for 5 min at 37 ^o^C. This was then followed by perfusion with Tyrode’s buffer containing 200 µM [Ca^2+^]_o_, 1.0 mg/ml Type II Collagenase (Worthington, NJ, USA) and 0.1 mg/ml Type I Protease (Sigma, MO, USA). The end of digestion was detected as a fast flow rate of enzyme solution in the form of a string coming from the coronary circulation. Ventricles were then dissected, diced into smaller blocks of ~ 1mm wide and triturated within Tyrode’s buffer containing 0.15 mM [Ca^2+^]_o_ to release single myocytes. The resulting suspension was collected in 50 ml falcon tubes and samples of the myocytes obtained were examined under the light microscope. The myocytes were fixed with 2% paraformaldehyde (PFA) at T_room_ for 10 min and washed for a further 10 min in phosphate-buffered saline (PBS) [2] .

*2.2 Reagents and chemicals*

A Tyrode’s solution was used containing (mM): 140 NaCl; 10 HEPES; 10 D-Glucose; 4 KCl; MgCl_2_; pH = 7.4 adjusted with NaOH for myocyte isolation experiments. Rabbit polyclonal to ARL13B (Proteintech, USA) and Mouse monoclonal to acetylated α-tubulin (clone 6-11 B-1, Sigma-Aldrich) antibodies were used to label primary cilia. Phosphate-buffered saline (PBS) (Gibco, Life Technologies, NZ) and PFA (8% aqueous solution, Electron Microscopy Sciences, Hatfield, PA) was diluted to 2% with PBS were available.

*2.3 Immunolabelling of primary cilia in rat tissue sections and isolated cardiomyocytes*

Rat hearts from different developmental stages - embryonic, neonatal (P2), young (P21 & P28) and adult (~ 2months) were investigated. Hearts were excised from anaesthetized rats after decapitation, washed with PBS, and fixed in 2% PFA for 30 min at room temperature (T_room_). Tissues were cryoprotected sequentially with 10%, 20%, and 30% sucrose solutions and stored at -80 ^o^C. Ten µm sections from neonatal (n = 3), P2 (n = 2), P21 (n = 3), P28 (n = 3) and adult rat hearts (n = 4) were rehydrated with PBS and were blocked with 10 % goat serum in PBS (1h at T_room_). Followed by overnight incubation with mouse monoclonal antibody to acetylated α-tubulin (1:500, diluted in 5% normal goat serum with 0.1% Triton X-100 in PBS). Next day, sections were washed in PBS followed by 2h incubation with goat anti-mouse Alexa Fluor 594 (1:500) at T_room_. In a separate experiment, sections were labelled with rabbit polyconal ARL13B (1:500) and goat anti-rabbit Alexa Fluor 488 (1:200) in similar manner as described above. All sections were also counterstained with Hoechst (1:200) for 10 min, washed in PBS and mounted in Prolong Gold antifade reagent.

Immunofluorescence of selected tissue sections triple labelled with antibodies for primary cilia (ARL13B), endothelial cells (RECA-1) / fibroblasts (vimentin) / f-actin (phalloidin), and nuclei (Hoechst) was carried out. Results showed ARL13B labelling was most closely associated with phalloidin labelling characteristic of cardiac myocytes (Figure 4C).

*2.3 Imaging*

Images (1024 x 1024 pixels) were acquired on an Olympus FV1000 confocal microscope (60x NA 1.35 oil-immersion objective). Alexa 488, 594 and 694 fluorochromes were excited with 473, 559, and 635 nm lasers respectively. Z-series were acquired with z slice thickness of 0.3 µm, and images with a sampling speed of 8.0 µs / pixel. The images were processed using Olympus Fluoview™ Version 3.0 Viewer and Image J (<http://imagej.nih.gov/ij>).

**Figure Legends**

**Figure S1. Controls for acetylated α-tubulin and ARL13B antibodies**.

**(i)** Confocal images of rat kidney sections taken from glomerulus and tubule regions. **(A)** negative, and **(B)** positive controls for acetylated-α-tubulin**. (C)** negative, and **(D)** positive controls for ARL13B. The images show the presence of primary cilia in the glomerulus region of the kidney. Scale bars are shown for each image.

**(ii)** A video of Z-stacks from the rat kidney glomerulus region stained with acetylated-α-tubulin as a positive control show primary cilia in different planes.

**Figure S2. Acetylated-α-tubulin labelling in adult cardiac tissue.**

A series of Z-stacks showing intracellular and extracellular tubulin labelling by acetylated α-tubulin (red) in adult rat cardiac tissue. Regions of intense staining are shown in the extracellular space, along with some less prominent intracellular staining of microtubules, illustrating the inappropriateness of acetylated-α-tubulin for identification of primary cilia in the heart. The scale bar is 10 µm.

**References**

1. Cooper PJ, Soeller C, Cannell MB. Excitation-contraction coupling in human heart failure examined by action potential clamp in rat cardiac myocytes. J Mol Cell Cardiol. 2010;49:911–7.

2. Soeller C, Jayasinghe ID, Li P, Holden A V, Cannell MB. Three-dimensional high-resolution imaging of cardiac proteins to construct models of intracellular Ca^2+^ signalling in rat ventricular myocytes. Exp Physiol. 2009;94:496–508.
