# Supplementary figures and images for: Evidence of primary cilia in the developing rat heart
Source: Cilia. 2018 Jul 31;7:4. doi: 10.1186/s13630-018-0058-z (PMC6069708; doi:10.1186/s13630-018-0058-z)

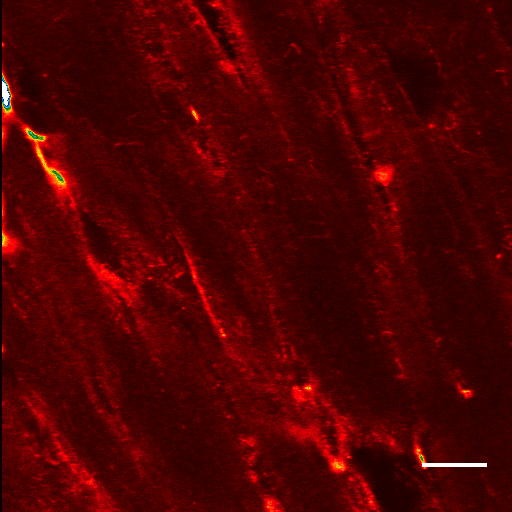

Supplement: Supplementary file 2 — Additional file 2. Acetylated-α-tubulin labelling in adult cardiac tissue. A series of Z-stacks showing intracellular and extracellular tubulin labelling by acetylated α-tubulin (red) in adult rat cardiac tissue. Regions of intense staining are shown in the extracellular space, along with some less prominent intracellular staining of microtubules, illustrating the inappropriateness of acetylated-α-tubulin for identification of primary cilia in the heart. The scale bar is 10 µm. [file 13630_2018_58_MOESM2_ESM.tif]

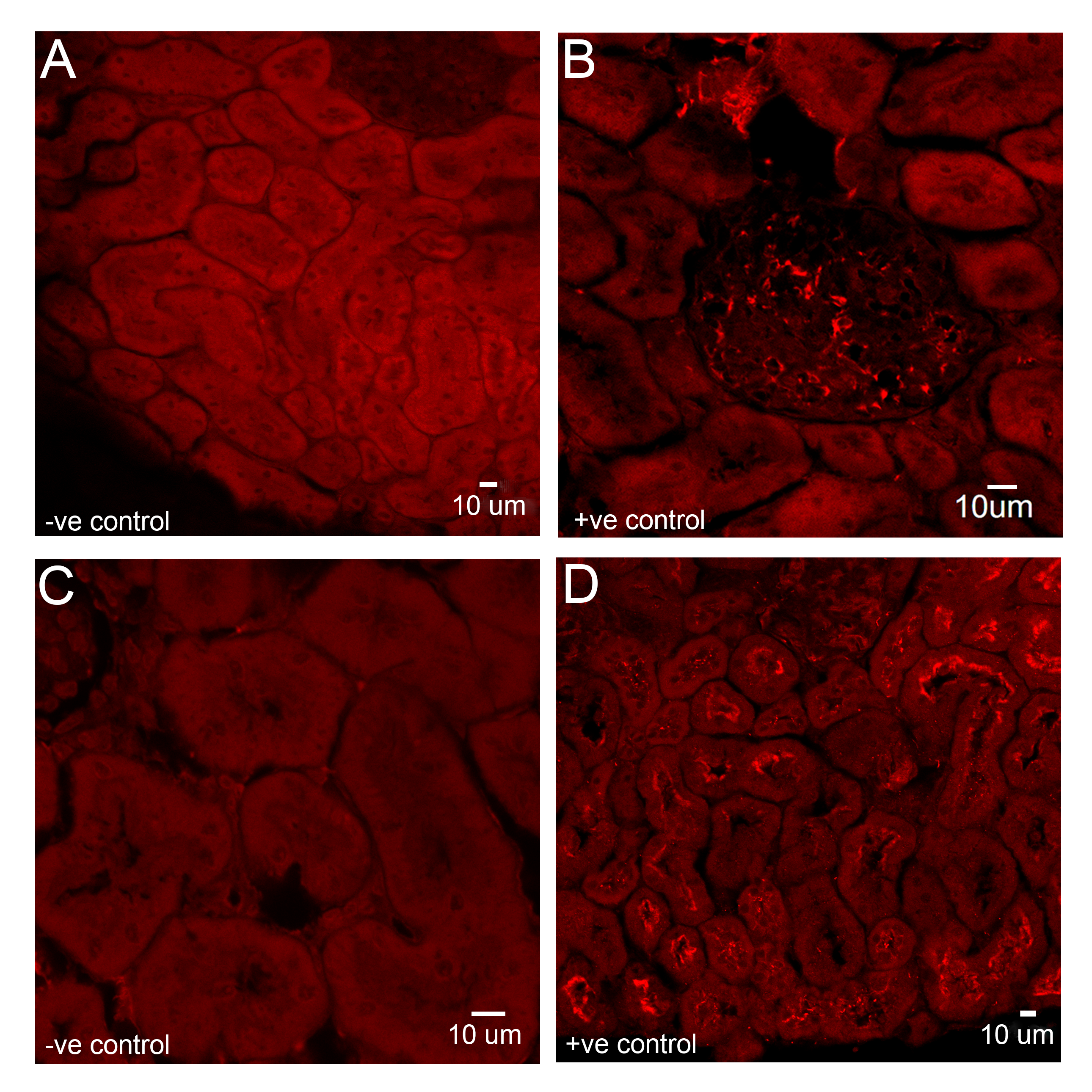

Supplement: Supplementary file 3 — Additional file 3. Controls for acetylated α-tubulin and ARL13B antibodies. (i) Confocal images of rat kidney sections taken from glomerulus and tubule regions. A Negative, and B positive controls for acetylated-α-tubulin. C Negative, and D positive controls for ARL13B. The images show the presence of primary cilia in the glomerulus region of the kidney. Scale bars are shown for each image. (ii) A video of Z-stacks from the rat kidney glomerulus region stained with acetylated-α-tubulin as a positive control show primary cilia in different planes. [file 13630_2018_58_MOESM3_ESM.zip › Figure S1(i).tif]
